# Alzheimer, dementia and the living will: a proposal

**DOI:** 10.1007/s11019-014-9559-8

**Published:** 2014-04-16

**Authors:** Claudia Burlá, Guilhermina Rego, Rui Nunes

**Affiliations:** 1 Palliative Care of the Federal Council of Medicine, Brasília, Brazil; 2 Department of Social Sciences and Health, Faculty of Medicine, University of Porto, Estrada da Circunvalação 9925, 4250-150 Porto, Portugal

**Keywords:** Advance directives, Alzheimer’s disease, Durable power of attorney, Genetics, Living will

## Abstract

The world population aged significantly over the twentieth century, leading to an increase in the number of individuals presenting progressive, incapacitating, incurable chronic-degenerative diseases. Advances in medicine to prolong life prompted the establishment of instruments to ensure their self-determination, namely the living will, which allows for an informed person to refuse a type of treatment considered unacceptable according to their set of values. From the knowledge on the progression of Alzheimer disease, it is possible to plan the medical care, even though there is still no treatment available. Irreversible cognitive incapacity underlines the unrelenting loss of autonomy of the demented individual. Such a loss requires the provision of specific and permanent care. Major ethical issues are at stake in the physician–patient–family relationship, even when dementia is still at an early stage. The authors suggest that for an adequate health care planning in Alzheimer disease the living will can be presented to the patient in the early days of their geriatric care, as soon as the clinical, metabolic or even genetic diagnosis is accomplished. They also suggest that the appointment of a health care proxy should be done when the person is still in full enjoyment of his cognitive ability, and that the existence and scope of advance directives should be conveyed to any patient in the early stages of the disease. It follows that ethical guidelines should exist so that neurologists as well as other physicians that deal with these patients should discuss these issues as soon as possible after a diagnosis is reached.

The improvements in health care and general living conditions that occurred throughout the twentieth century in developed nations contributed to a longer and healthier life. The United Nations Report World Population Ageing points out to projections of two thousand million people aged 60 and over by 2050, imposing a series of social challenges (United Nations 2010a, b). The euphoria of longer life expectation is counteracted with the problems that ageing carries, especially in the health area.

The advancement of medical intervention practices to maintain and prolong the lives of people in a state of chronic and sometimes terminal illness has prompted the creation of advance directives that emerged four decades ago with the purpose of enabling an informed person to refuse certain types of treatment which, according to their values, are unacceptable (Perkins 2007). By advance health care directives, or advance directives, it is meant both the living will in the traditional sense—a written document available in paper or in the health care system intranet (when it is technically possible), where the autonomous person makes choices with regard the treatments or other interventions that he wishes or not for himself—as well as the durable power of attorney for health care. The durable power of attorney makes it possible for an autonomous person to appoint someone he trusts (health-care proxy or surrogate) to make any necessary health care decisions in accordance with the substituted judgment approach when he is incompetent to decide. Therefore throughout this article the expression “advance directive” will be used interchangeably with “living will” insofar as the written document is concerned.

A matter of concern is the high prevalence of dementia in the very elderly people. Dementia is chronic and progressive and affects several brain functions, including memory, thinking, orientation, calculation, learning capacity, language and judgment (American Psychiatric Association 1994). The deficits in cognitive function are commonly accompanied, and occasionally preceded by deterioration in emotional control, social behaviour or motivation (Starr 2010). The most common cause of dementia is Alzheimer’s disease accounting for 60–70 % of cases (World Alzheimer’s Report 2009). Although Alzheimer Disease is a form of dementia there are other syndromes that have similar symptoms—such of depression, hallucinations, memory loss—syndromes that include dementia of Lewy bodies, vascular dementia, frontotemporal dementia, etc. (Farlow 2010). However, and notwithstanding the fact that the ethical background has some similarities, this article will focus exclusively on Alzheimer disease.

Also, recent scientific findings determined that mild cognitive impairment (MCI) can be detected more than 10 years before full diagnosis and that amyloid-β peptide deposits can be detected by amyloid imaging even earlier (Jack et al. 2011). Because the changes caused by MCI are not severe enough to affect daily life, the patient does not meet diagnostic guidelines for dementia.

Although significant research has already been performed with regard the diagnosis and treatment of currently incurable neurodegenerative dementias such as Alzheimer’s, for the time being it is still considered as an incurable disease. The discovery of genes responsible for early-onset Alzheimer’s dementia will not only make early diagnosis and treatment of the disease possible, before brain damage occurs, but can also lead to the prediction of the disease through genetic technology (Nordgren 2010). It follows that when MCI is detected or when the genetic basis of this neurodegenerative disorder is acknowledged (Feero et al. 2010) steps might be taken to empower patients through an advance directive although the right not to be informed about the result of such a screen and the right to refuse an advance directive should always be respected. But the existence of advance directives should also promote research and development of new treatments and new technologies for dementia. It follows that in accordance with agreed ethical principles it is imperative to reach a balance between the interests of society—in promoting new treatment modalities for Alzheimer—and patients’ basic rights of self-determination and privacy.

The practice of medicine requires us to know in depth the clinical and pathological aspects of the different diseases that affect people; such knowledge, however, is insufficient if other areas of knowledge are not considered, such as those from the social sciences and humanities. Any disease becomes an illness and even a sickness when a specific set of symptoms affects the life and wellbeing of the patient. A global understanding of the illness and its impact in the personal biography means also that the values in which the patient/physician relationship is embedded are considered so that the best outcome is achieved. Indeed, the sick person’s autonomy is deeply rooted in the bioethical discourse as a principle, meaning self-determination, empowerment to ensure the self-determination and self-government of the sick person in decisions about the treatment that he should be given. Autonomy presupposes the lapidary principle of freedom of choice.

However, the scope of the health system still does not fully cover the patient’s autonomy, in the broad sense, especially those whose capacities are impaired. Respect for freedom of choice of the person is a goal directed to guiding the process of achievement that will provide the health system with the necessary bioethical support. To bring the ethical issue of autonomy to the dementia scenario is a challenge. When it occurs, the irreversible cognitive impairment attests to the inexorable loss of autonomy of the elderly that suffer from Alzheimer disease. This loss implies the need for exclusive and permanent care. Although it is not clear at all whether the proxies have such an ethical responsibility the authors suggest that family members, taking into account the possible preservation of the sick person’s autonomy, have the ethical responsibility to perceive and realize what would be the will of the patient with dementia (Smith et al. 2013; Peter et al. 2011).This is a difficult task for a relationship in which cognitive asymmetry imposes itself radically.

This irreversible loss of autonomy is a challenge to the person and to the family. It should be reminded, though, that there is frequently a long mild-dementia stage, when a person’s autonomy comes and goes. In this situation good ethical practice determines that the expressed wish of the patient with Alzheimer has precedence over a written document or the will of the health-care proxy. Indeed, from the personal perspective most people praise autonomy not only because it is the only way to develop one’s talents and capacities, and therefore to be a full rational being, but also because no one likes to be dependent on others specially if the person has a story of a lifelong trajectory in pursuing his goals with independence and liberty. It follows that the mere prediction of depending on others is troublesome to many people and can even anticipate an important clinical decline. From a familial perspective the irreversible loss of autonomy is also a challenge because the image and identity of the person is deeply changed and this “new” person is sometimes dissociated with the familial biography. Moreover, the loss of autonomy usually implies a deep burden to the family and even to society, a circumstance most people find troublesome. Therefore, and although all lives are worth living, in the Alzheimer scenario the irreversible loss of autonomy due to the decline in mental functioning is considered by many people as a unwanted condition that could be minimized by the prospective use of the living will. But it should be emphasized that the will of a person can change with time and that the “new” person’s autonomy can be different from the previous living will. If that should be the case it should be given the patient with Alzheimer the opportunity to express his wishes and even to determine that the living will is not an option any more.

The objective of this paper is to approach advance directives as one of the tools for an adequate advance care planning in Alzheimer’s disease. This theme is of utmost importance due to, on the one hand, the demographic evolution of contemporary societies and, on the other, the recent approval in many countries of laws that regulate advance directives, such as the living will and the durable power of attorney. The article will also discuss the problems regarding the right time to recommend advance directives as an instrument for preserving and enhancing autonomy of the elderly with Alzheimer’s disease.

## Loss of autonomy of the person with Alzheimer’s disease

In April 2012, the World Health Organization (WHO) published the document “Dementia: A Public Health Priority” (World Health Organization 2012) demonstrating the seriousness of this problem that affects the quality of life of elderly individuals worldwide. Projections of incidence and prevalence indicate continued growth in the number of people with dementia, especially among the very old. By “very old” it is meant technically the “oldest-old” that is people aged 85 or older (United Nations 2010a, b). This report estimates at 35.6 million the number of individuals with dementia in 2010, and forecasts that this number will double every 20 years, i.e. it will be 65.7 million in 2030 and 115,4 million in 2050 (Camicioli and Rockwood 2010). However, to live longer implies the physiological decline of bodily functions and, consequently, increasing the number of individuals with chronic-degenerative diseases that are disabling, progressive, involutive and incurable diseases. Diseases previously considered fatal now acquire a chronic character, compatible with life (Ames 2005). The advanced age associated with high prevalence of chronic diseases may compromise the individual autonomy of many people. Typical examples are the dementia syndromes which find in age their greatest risk factor. Dementia is sometimes devastating not only for the people who suffer from it, but also for their caregivers and family. This is why the WHO program of action on mental health included dementia in a group of diseases that deserve priority attention (World Health Organization 2010).

Alzheimer’s disease is usually a slow progressing one and can affect individuals in different ways (Clearly et al. 2005). As the disease evolves, the deterioration is progressive and people experience difficulties in their daily lives, which makes them dependent on help for simple day to day tasks. In the advanced stage, in addition to the impairment of long term memory, there is the need for supervision for basic activities such as bathing, dressing, going to the toilet, eating and other daily activities. In the final stage of the disease, the person loses the ability to communicate, no longer recognises family and friends, becomes bedridden and dependent 7 days a week (Alzheimer’s Disease International 2010).

Currently, it is possible, through the knowledge acquired about the evolutionary course of Alzheimer’s Disease, to plan with regard to medical care, social support, financial and legal aspects, even though there is no medical treatment that can stop or reverse the course of the disease. Formal recognition of the rights of people with dementia through legislation and regulatory processes will help reduce discriminatory practices and thus ensure care and protection measures in the advanced stage of the disease, where the capacity for judgment and self-determination are impaired, precluding control over their own decisions. In many circumstances these rights are already guaranteed given the ethical requirements for nursing and palliative care. But nevertheless rights of self-determination could be extended if the professional ethics of physicians and nurses is complemented with tools that allow a rational and informed decision-making process of the prospective patient with Alzheimer.

Semantically, the word autonomy comes from the Greek autos which means “self”, and nomos which means “sharing”, “law of sharing”, “institution”, “use”, “law”, “convention”.

Autonomy consists of self-government, in manifestations of subjectivity, in making their own laws that will guide their life and in the Kantian tradition persons’ law-making define laws that can be universalised for the general society, i.e. for any person. It means the recognition of free, rational, uncoerced individual choice about their own interests whenever it does not affect third-party interests. The autonomy of the person presupposes respect for the right to decide about his life, regarded as an absolute condition of human freedom (Engelhardt 1996). In the health care context, the core of the concept of autonomy has been linked to the exercise of self-determination and it is closely connected to quality of life. One of the ways to evaluate the quality of life of a person is to consider the degree of autonomy that he has, taking into account the socio-cultural context in which he lives (Miranda et al. 2009).

In 1979 the “Belmont Report” established the fundamental ethical principles to guide research with human subjects. The word autonomy was definitely incorporated into biomedicine, meaning a human competence in which the patient is permitted to define his own decisions, regardless of other powers, for the self-determination to make decisions about his medical treatment (National Commission for the Protection of Human Subjects of Biomedical and Behavioral Research 1979). With regard to the patient’s autonomy in making decisions about the health care that he should or should not be submitted to, Beauchamp and Childress offer important contributions in the model called pure autonomy: meaning that patients that are already unable to decide about themselves, but who, when they were autonomous, expressed a preference or relevant decision, will now have guidance on the decision-making process about their care (Beauchamp and Childress 2012).

In a situation of irreversibility in which the person has a disease that evolves into a terminal state, one way to preserve autonomy is the right to express him as to which treatments he should or should not be submitted to. In this case the existence of an advance directive might ensure his determinations and wishes when the person is no longer in a condition to do so by himself. Just as in the end of life (Johnson 2005), the situation of patients with Alzheimer’s disease is challenging. The irreversible brain damage that may happen will gradually destroy the independence of these patients and make them dependent. In this case, the cognitive impairment prevents them from exercising their autonomy to make their own choices. It is a peculiar situation, where ethical implications arise related to the complexity of human relations, aggravated by a radical asymmetric interaction, in which a participant determines and the other submits. But it can also be argued that in dementia preservation of autonomy does not lead necessarily to the living will or to the appointment of a durable power of attorney because respect for self-determination can have different meanings in different settings.

Human beings are not born autonomous; only their development in the course of time enables them to create their own guidelines and be guided by them; this freedom is based on ensuring a comprehensive education of the will and opinion (Jacques 1965). Alzheimer’s disease, by causing a progressive loss of autonomy, creates a condition of vulnerability that could compromise the rights of the person (McKhann et al. 2011). According to Rigaux even his dignity could be at stake if the patient with Alzheimer is not respected as a full human person (Rigaux 2011). Advance directives can be a useful tool for the medical decision-making process both in people who have an early on-set dementia (before 65) and in those who are older, or even much older. Older people with Alzheimer’s disease might have an additionally chronic disease or diseases (multi-morbidity), such as diabetes or heart failure (which also could affect the cognitive level). Therefore a question that could be asked is which disease/illness/functional decline would take precedence over the others in the context of an advance directive? In this setting the relevant issue is not only a question of the nature of the disease but the degree of cognitive impairment. It follows that when MCI arrives, this might be considered the starting point for the discussion over the purpose and scope of an advance directive. In this way whatever the comorbidities of the Alzheimer patient it should be clearly determined if there exists, or not, enough capacity to decide autonomously.

On the other hand, some patients with MCI never develop Alzheimer’s disease. So to be ethical the physician should proceed with extreme caution not to impose an advance directive but only to expose its existence and usefulness. Also, the living will might not be regarded as a one-off-event (when the person is cognitive intact) but as a process even far into the disease. Advanced stages of the disease might enable the patient to make some decisions in proportion to his mental capacity. There is of course an ethical line after which no competence exists in some patients with dementia and therefore offering a living will is not an option any more.

## The challenge of the living will in Alzheimer’s disease

The course of Alzheimer’s disease until the end of life might challenge the patient’s ability to manage and control his deeds, wishes and even to make choices. This frail individual, dependent and unable to express his own will, loses his power to decide and resolve. He is thus totally dependent on his family members, caregivers, professionals or those who are closest to him (Prince et al. 2011). The Universal Declaration on Bioethics and Human Rights, approved by UNESCO in October 2005, presents the ethical principle of protection of the vulnerable individuals, emphasizing respect for their autonomy. The patient with Alzheimer is potentially vulnerable without perspective of reversal, which makes appropriate information and guidance even more necessary. The questions are unsettling: is the patient with dementia excluded from the possibility of exercising the freedom of choice of his treatments? At what point should the living will be proposed to him so that he can fully express his wishes? Advance directives can be an option to minimize the prospective loss of autonomy.

Doctor, patient and family are subjects of decisions that result in relevant guidelines and available treatments. It follows that when the Alzheimer patient begins to lose autonomy the sharing of decisions might be an option that should be overtly discussed with him (Murray and Jennings 2005). Informed consent is a usual practice in health care, although this in itself does not always achieve effective communication dynamics. In this context, doctor-patient-families are in a relational praxis. The anguish suffered with the disease, the perplexity of the unknown future can sometimes hinder or even derail this communication, especially with family members.

One way to preserve autonomy is the right to express him as to which treatments he should or should not be submitted to. In this case the existence of previously expressed wishes, and a document like the living will might ensure his determinations and wishes when the person is no longer in a condition to do so by himself. As we shall see both the living will as the durable power of attorney will not resolve all ethical disputes in the clinical setting. But as they promote an honest discussion between the patient, the family and healthcare providers it may increase communication between all parties involved.

Major ethical problems are involved in the relationship doctor-patient-family even when Alzheimer’s disease is still at an early stage of evolution. The guideline for the preparation of the living will presupposes that the person is lucid, conscious and with full autonomy to record his decisions for the time when he cannot speak for himself. It might also include the appointment of a legal representative so that his decisions are complied with. Indeed, the living will is a written statement that details the type of care a person wants (or do not want) if he becomes incapacitated.

The durable power of attorney for health care is applicable whenever the person is competent to do so. Preferably one should appoint the health care proxy in the absence of disease and Alzheimer’s disease is no exception. However, as this argument goes the health care proxy can still be appointed in the early stages of dementia. For instance when it is detected genetically, by brain amyloid imaging technology or even when mild clinical symptoms emerge. Therefore, if an advance directive is an option (the appointment of a health care proxy and/or a living will) it is critical the moment it is introduced.

In some countries, such as the United States, many people aged 65 or more already have an advance directive (65 % of nursing home residents according to Adrienne Jones et al. 2011) because in the last 20 years it is legally required in most health care facilities to inform adult patients about their rights to execute an advance directive. However, in many countries where such laws do not exist the prevalence of advance directive among the elderly is much lower.

It is up to the attending physician to suggest this course of action in the very early stages of dementia as well as to determine, in a particular circumstance, if such a person is still autonomous to make an informed decision. Evaluating the patient capacity to decide for him is a complex task and sometimes consulting with other professionals is necessary to determine the patient’s agency. The disease stage and its impact in the will of the patient with Alzheimer are a determinant and a predictor of an adequate ethical outcome. It follows that the advance directive can be executed before, during or immediately after the diagnosis of dementia as long as adequate competency (and therefore autonomy) is still preserved. As a guideline for the healthcare providers it should be emphasised that the possibility of an advance directive should be offered to the patient with Alzheimer as soon as it is a possibility. When for many different reasons, this is not possible the advance directive should be suggested in any stage of the disease compatible with a rational and autonomous decision. It should be emphasized that the precise moment when an advance directive is executed is of utmost importance to determine its ethical acceptance, notwithstanding the fact that the practical use of an advance directive can be anywhere in the future.

Indeed, according to Perkins (2007) there are two different characteristics of the living will: contribution to patient empowerment and to self-determination; and facilitating the advance care planning, meaning an adequate planning of the moment of death since that, for many different reasons, this issue is frequently ignored by many people and by many health professionals. That is, the conditions are in place so that the patient, in a very preliminary phase of Alzheimer’s disease, can make choices on health, either on the treatments that he wants or does not want to receive or on the appointment of the one who can best represent him (durable power of attorney for health care) in the foreseeable situation of incapacity to do so. And, the doctor, having ensured a good doctor–patient–family relationship, would be the professional of choice to introduce the possibility of the living will. If an advance directive is at stake the moment it is suggested to the patient with Alzheimer is critical because after losing competence the patient no longer has the cognitive conditions to exercise the right to his autonomy.

A significant number of elderly people—at least one in four—need someone to make decisions about their medical care at the end of life. This circumstance illustrates the importance of people registering their wishes in life and/or designating someone to make decisions regarding their medical treatment. This is the only way for people who formally stated their preferences in specific documents to have the treatment they want.

It is well known that people who has an advance directive is more likely to want limited care than to receive all possible assistance (Silveira et al. 2010). Indeed, although an advance directive can be used for expressing wishes over treatments that a person wants or does not want it is more likely to be used to limit care, and for different reasons. First of all the perception, sometimes wrong, that there are no defined limits to withholding and withdrawing life-prolonging medical treatment in terminal and chronic patients, namely futile treatments, sedation for refractory symptoms of terminal patients that may hasten death, or even decisions to forego medical treatment in the permanent vegetative status. The living will might allow for an easier withdrawal or withholding of futile treatments giving a sense of control that is usually felt as an opportunity to alleviate pain and suffering. On the other hand many people, namely when facing a diagnosis of Alzheimer, feel that they do not want to be a burden on their family and society. Therefore, elderly people who have prepared a living will, in general, receive the care strongly associated with their preferences.

These claims strongly suggest the formulation of the living will when individuals are still competent to decide or at least that the patient with Alzheimer is informed of the possibilities of advance directives either in the form of a living will or of a healthcare proxy. From an ethical perspective, though, the living will has precedence over the healthcare proxy because it is, at least in principle, more in accordance with the wishes of the patient. It has been observed that, at the present time, advance directives are an adequate instrument to respect the autonomy of the sick person.

A respect that is imposed against the inalienable freedom of the human being to decide about himself as well as the choices about the medical interventions that may be proposed. Thus, advance directives present itself as a significant advance in the area of health which has its origin in the person’s ethical freedom and is in accordance with the deep social transformations that enable its widespread acceptance. However, in the case of Alzheimer’s disease, one can radicalize the position of the doctor as suggested by Twycross (2002) between the arrogance learned from “I know what’s best for you” and the impossible delegation of “you should decide for yourself”. True to ethical principles, especially in defence of respect for patient autonomy the doctor himself is faced with the challenge of finding a way to keep his promise to comply with the principles embraced by medicine in the twenty first century, never forgetting that the patient must be properly informed in order to make an appropriate and informed decision, an essential premise of the ethics of advance directives. The general practitioner, as well as other health care professionals, is specially prepared to convey the scope and importance of the living will although in the future advance directives should be a part of the general health literacy.

That is, the preparation of a living will implies that the person is in possession of his full cognitive capabilities so that he can clearly see the scope of his decisions with regard to the possibility of effective choices by and for himself in health care. These aspects can be articulated in a proposal that gives the patient with Alzheimer a new perspective for the future:

- First, the existence and scope of advance directives should be conveyed to any patient in the early stages of Alzheimer’ disease. It follows that ethical guidelines should exist so that physicians as well as other professionals that deal with these patients should discuss this issue as soon as possible after a diagnosis is reached.
- Second, the appointment of a health care proxy who, preferably, is elected by the person in full enjoyment of his cognitive ability, implies that he knows reasonably well the axiological biography of the patient so that any decision is an informed one in accordance with the desires and expectations of the patient. This proxy must respect the patient’s legitimate right to self-determination, and so he will ensure what would be the wishes of the person with Alzheimer’s disease (substituted judgement). It should be pointed out that if during the treatment the patient is still autonomous and disagrees with the health care proxy’s approach (namely to follow the previously defined living will or the proxy’s judgement) the will of the patient always prevails. Also, that only the person and no one else can appoint a health care proxy because not only is the proxy someone of trust but also someone that deeply knows the values embraced by the patient.
- Finally, at a state level, countries should promote as a matter of public policy the creation of a network within the health system so that the living will and the durable power of attorney are immediately available on-line (if this is the wish of the person). It implies that necessary precautions are taken so that privacy rights are not violated due to an unauthorised access to this information. Special precautions should be taken to avoid abuse of privileged information namely the identity of the person, and other biographical data, as well as classified information about the previous wishes of the patient. From a professional perspective this should also be considered as an ethical imperative strongly regulated by ethical codes.

## Conclusion

In some stages of Alzheimer’s disease there is no possibility to reliably obtain information on the person’s wishes, since his cognitive ability is compromised. Any opinion of the relatives and the professionals involved, even if well intentioned, does not necessarily express the wishes of the patient. This conflict could only be clarified if there had been a previously expressed record. And for this, the living will must be prepared by the patient with Alzheimer before the onset of dementia. Or, alternatively, immediately after the clinical diagnosis is made by the doctor. Indeed, MCI causes cognitive changes that are serious enough to be noticed by the individuals experiencing them or to other people and its detection should be followed by an honest discussion about the benefits and limits of advance directives.

In the universe of suffering in Alzheimer’s disease, it is recommended that humanitarian attitudes and conduct, regarding patient care, should prevail in the doctor-patient-family relationship. However, it is observed that this scenario often leads to conflict and disagreements that can transform solidarity into solitariness, either on the part of the family or the doctor himself. Respect for the dignity of the human person is the primary concern to encourage patients with Alzheimer to exercise autonomy and to document their wishes in advance directives.
